# Supplementary material for: 14-3-3 binding maintains the Parkinson’s associated kinase LRRK2 in an inactive state
Source: Nat Commun. 2025 Aug 5;16:7226. doi: 10.1038/s41467-025-62337-1 (PMC12325948; doi:10.1038/s41467-025-62337-1)
Supplement: Supplementary file 1 — Supplementary Information [file 41467_2025_62337_MOESM1_ESM.pdf]

Supplementary Materials for:

14-3-3 binding maintains the Parkinson's associated kinase LRRK2 in an inactive state

Juliana A. Martinez Fiesco[1], Alexandra Beilina[2], Astrid Alvarez de la Cruz[1], Ning Li[1], Riley D. Metcalfe[1], Mark R. Cookson[2], Ping Zhang[1*]

[1]Kinase Complexes Section, Center for Structural Biology, Center for Cancer Research, National Cancer Institute; Frederick, MD, 21702, USA.

[2]Cell Biology and Gene Expression Section, National Institute on Aging, National Institutes of Health, Bethesda, MD, 20892, USA.

*Corresponding author. Email: ping.zhang@nih.gov

The PDF includes:

Supplementary Figs. 1 to 14

Supplementary Tables 1 to 2

Other Supplementary Material for this manuscript includes the following:

Supplementary Movie 1

Source file

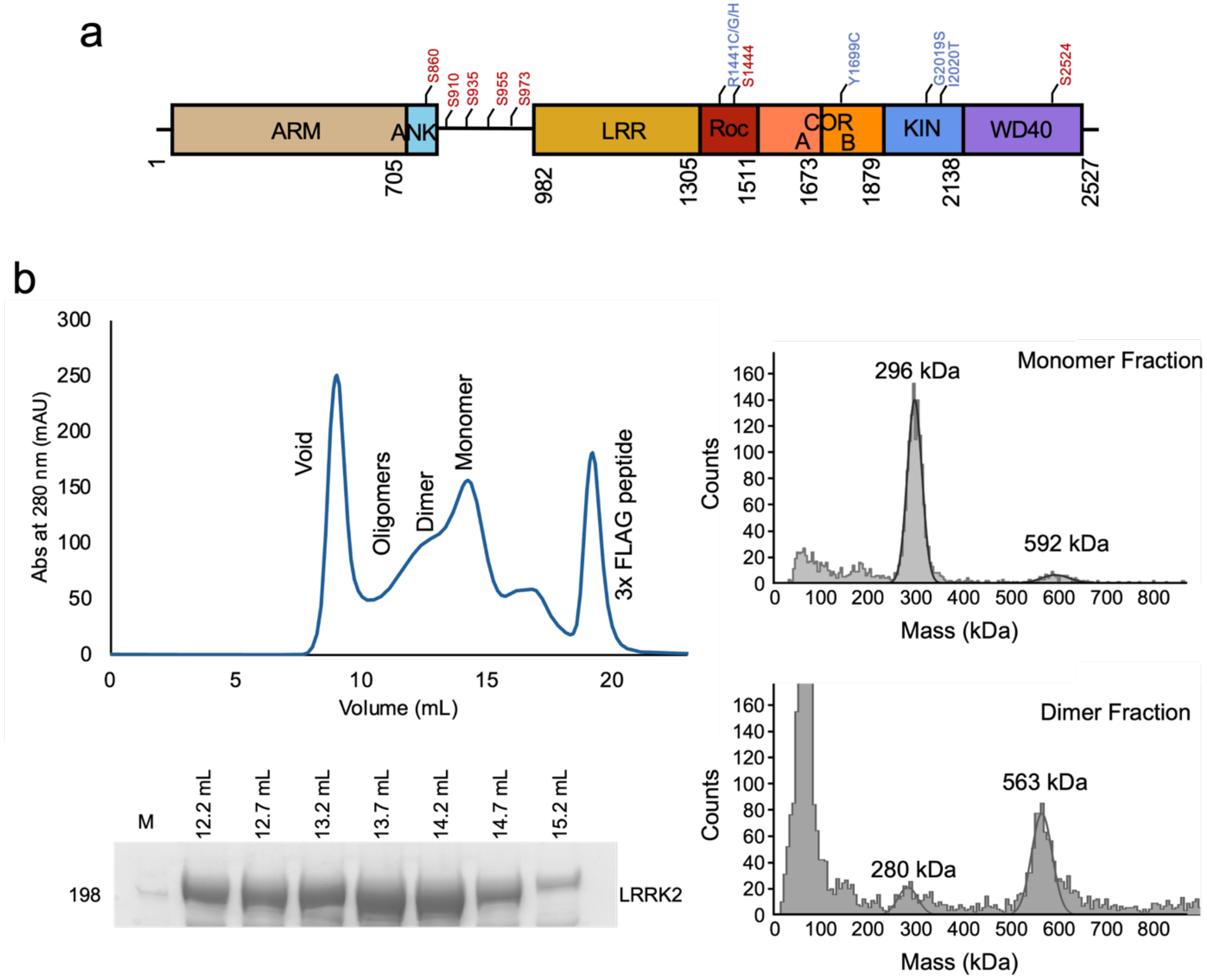

Supplementary Fig. 1. Isolation and characterization of human LRRK2.

a. Schematic of LRRK2 domain organization with potential 14-3-3 phospho-binding sites (marked in red) and prominent PD mutations (marked in blue). **b.** Purification and characterization of LRRK2. Size-exclusion chromatogram from a Superose 6 Increase 10/300 gel filtration column reveals distinct peaks corresponding to monomeric and dimeric LRRK2 (top left). The corresponding SDS-PAGE gel analysis of LRRK2 fractions collected during gel filtration (bottom left), along with mass photometry (right) of the monomer and dimer fractions, confirms their oligomeric states. Refer to source data for complete membrane images.

a

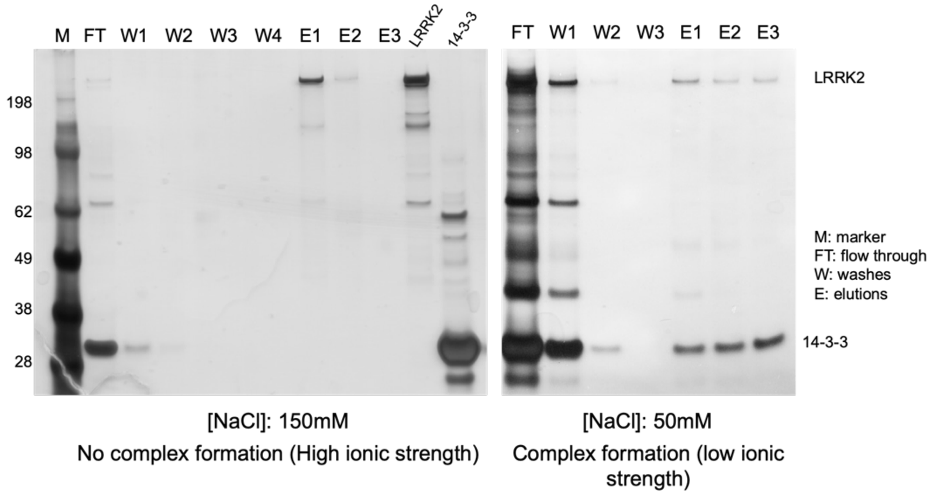

b

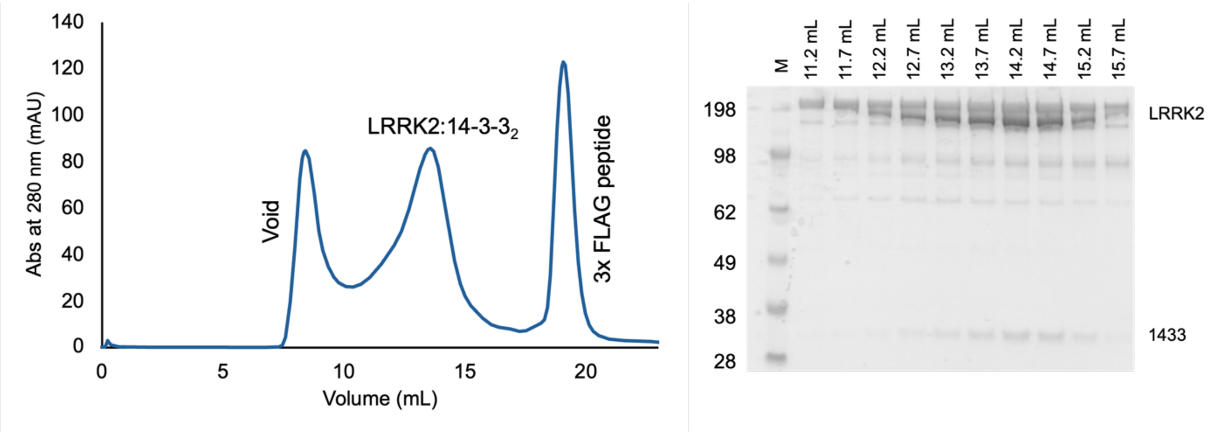

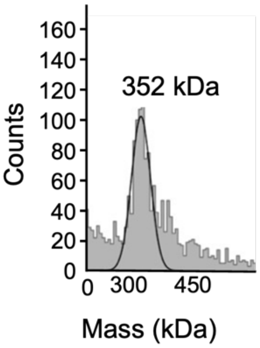

Accession/Gene	Protein	Coverage (%)	#PSMs	# Unique Peptides
Q5S007/LRRK2	Leucin-rich repeat serine/threonine-protein kinase 2	54%	1397	129
P61981/YWHAG	14-3-3 protein gamma	63%	161	15
P62258/YWHAE	14-3-3 protein epsilon	52%	44	10
Q04917/YWHAH	14-3-3 protein eta	17%	37	1
P27348/YWHAQ	14-3-3 protein theta	24%	35	2
P63104/YWHAZ	14-3-3 protein zeta/delta	19%	20	2

Supplementary Fig. 2. Isolation and characterization of human LRRK2/14-3-3 complex. a. Dependency of LRRK2/14-3-3 complex formation on the ionic strength of the solution. Co-IP assays using purified FLAG tagged LRRK2 to capture 14-3-3γ showed that LRRK2 does not form a stable complex with 14-3-3 at high salt concentration (left gel), while a stable complex is formed under low salt conditions (right gel), illustrating the ionic sensitivity of this interaction. **b.** Biochemical characterization of the LRRK2/14-3-3 complex. A gel filtration profile from a Superose 6 Increase 10/300 column (top left) indicates formation of the LRRK2:14-3-3_2 complex. SDS-PAGE analysis (top right) validates the co-elution of LRRK2 and 14-3-3 proteins in the corresponding peak fraction. Mass photometry measurements (bottom left) confirm the stoichiometry of the complex. Mass spectrometry analysis (bottom right) of the complex in solution is also shown, with the number of total (Peptide Spectrum Matches (PSMs)) and unique peptides reported. Refer to source data for complete membrane images.

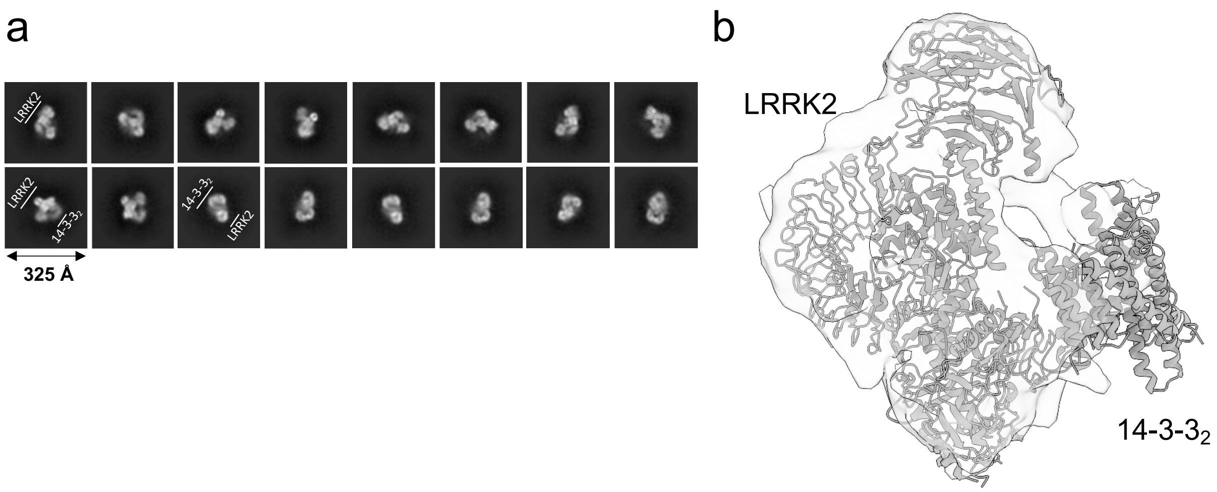

Supplementary Fig. 3. Diagnostic cryo-EM analysis of the LRRK2/14-3-3 complex. **a.** Representative 2D class averages of the LRRK2/14-3-3 complex. **b.** Cryo-EM density map of the complex at 9.4 Å resolution with the monomeric LRRK2 (PDB: 7LHW) and 14-3-3 dimer (PDB: 2B05) fitted in the map, illustrating the overall architecture of the complex.

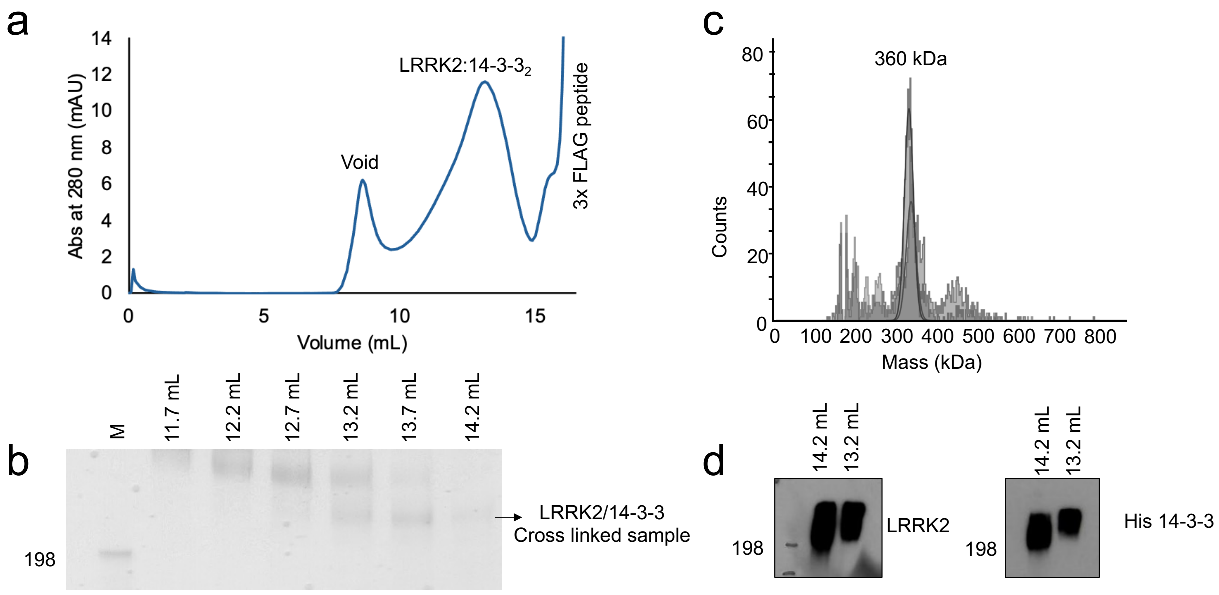

Supplementary Fig. 4. Purification and biochemical characterization of the BS3-cross-linked LRRK2:14-3-3$_2$ complex. a. Gel filtration chromatogram on a Superose 6 Increase 10/300 column showing the elution profile of the complex. **b.** 4-12% SDS-PAGE gel analysis indicating the presence of the cross-linked cross LRRK2/14-3-3 band. **c.** Mass photometry analysis depicting the mass distribution of the corresponding gel filtration LRRK2:14-3-3$_2$ fraction. **d.** Western blot confirming the cross-linking of FLAG-LRRK2 and His-14-3-3γ, using anti-LRRK2 (Abcam cat. ab133474) and anti-14-3-3γ (Abcam cat.137048) antibodies. Refer to source data for complete membrane images.

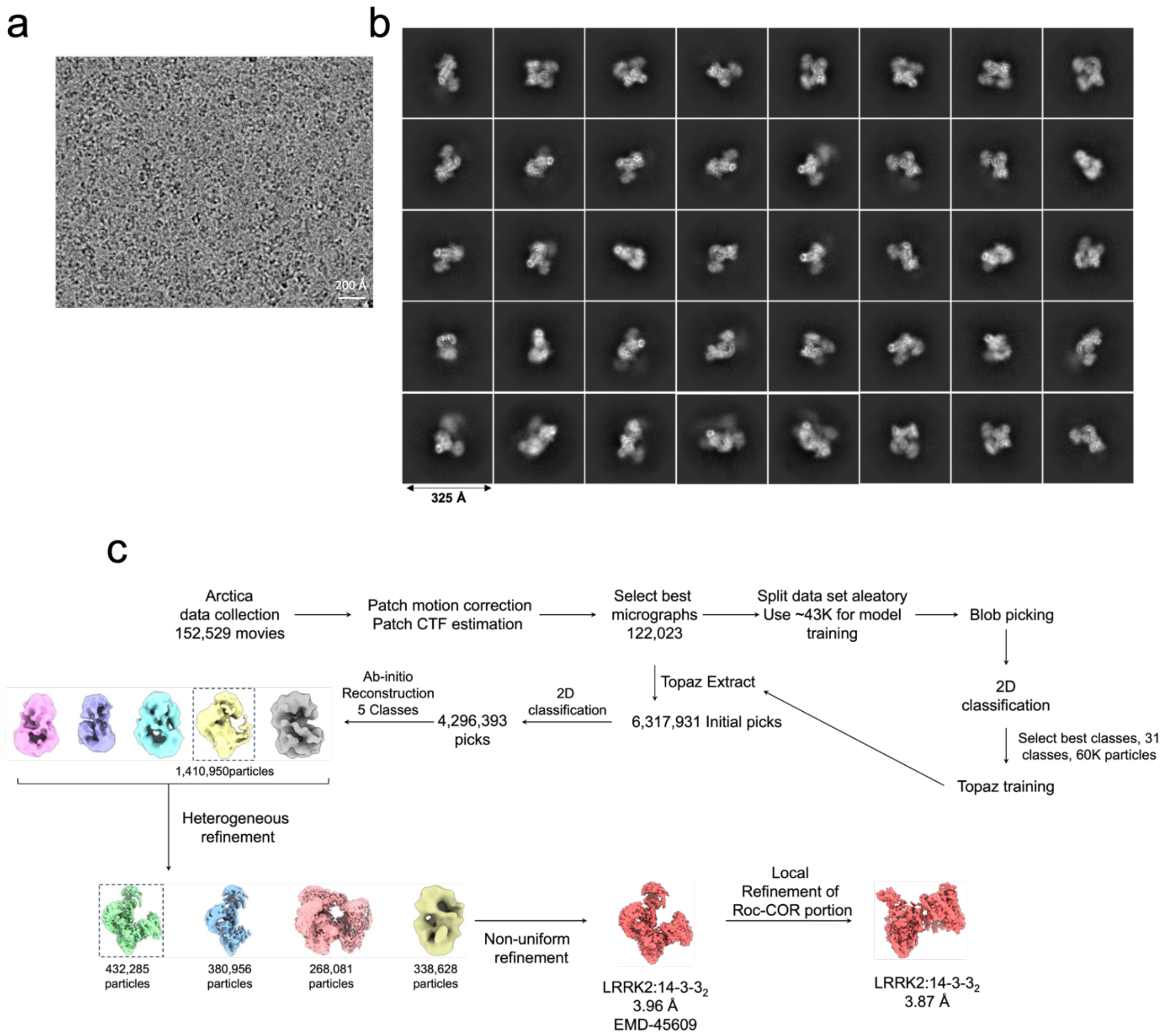

Supplementary Fig. 5. Cryo-EM data collection and processing of the LRRK2:14-3-3$_2$ complex. a. Representative cryo-EM micrograph. **b.** Selected 2D class averages of LRRK2:14-3-3$_2$ particles **c.** Schematic of the cryo-EM 3D reconstruction workflow.

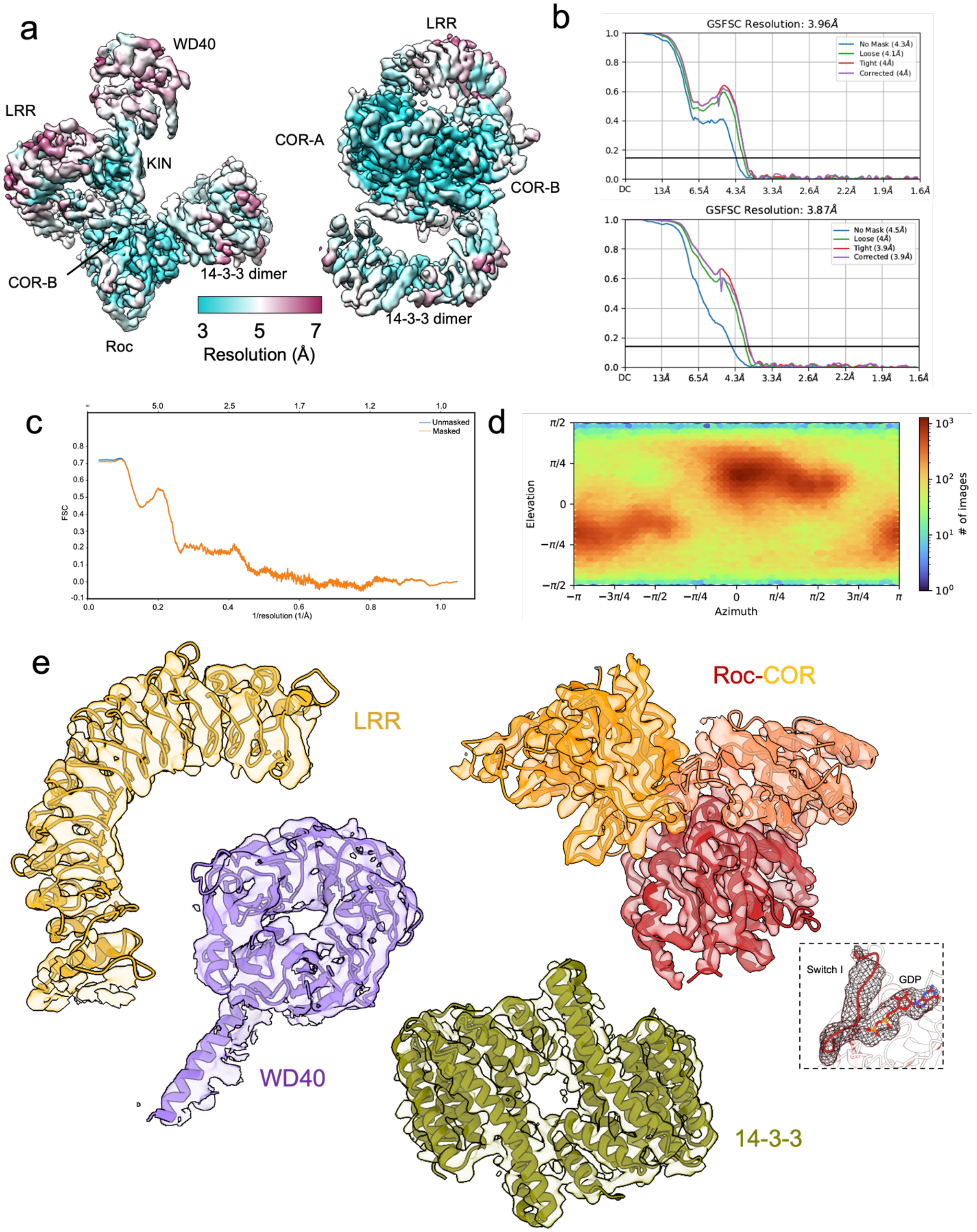
a
WD40
LRR
KIN
COR-B
14-3-3 dimer
Roc
3
5
7
Resolution (Å)
LRR
COR-A
COR-B
14-3-3 dimer
b
GSFSC Resolution: 3.96Å
No Mask (4.3Å)
Loose (4.1Å)
Tight (4Å)
Corrected (4Å)
GSFSC Resolution: 3.87Å
No Mask (4.5Å)
Loose (4Å)
Tight (3.9Å)
Corrected (3.9Å)
c
Unmasked
Masked
FSC
1/resolution (1/Å)
d
Elevation
Azimuth
# of images
e
LRR
Roc-COR
WD40
14-3-3
Switch I
GDP

Supplementary Fig. 6. Comprehensive cryo-EM analysis of the LRRK2:14-3-3$_2$ complex. a. Cryo-EM map displayed with color coding according to local resolution. **b.** Golden-standard Fourier shell correlation (FSC) curves for the resolution assessment of the global (top) and Roc-COR:14-3-3$_2$ local (bottom) resolution maps. **c.** Map-to-model FSC curve evaluating model fit to the cryo-EM density map. **d.** Angular distribution plot of particle orientations for the global non-uniform refinement. **e.** Detailed views of density regions of specific LRRK2 domains and 14-3-3 protein within the complex.

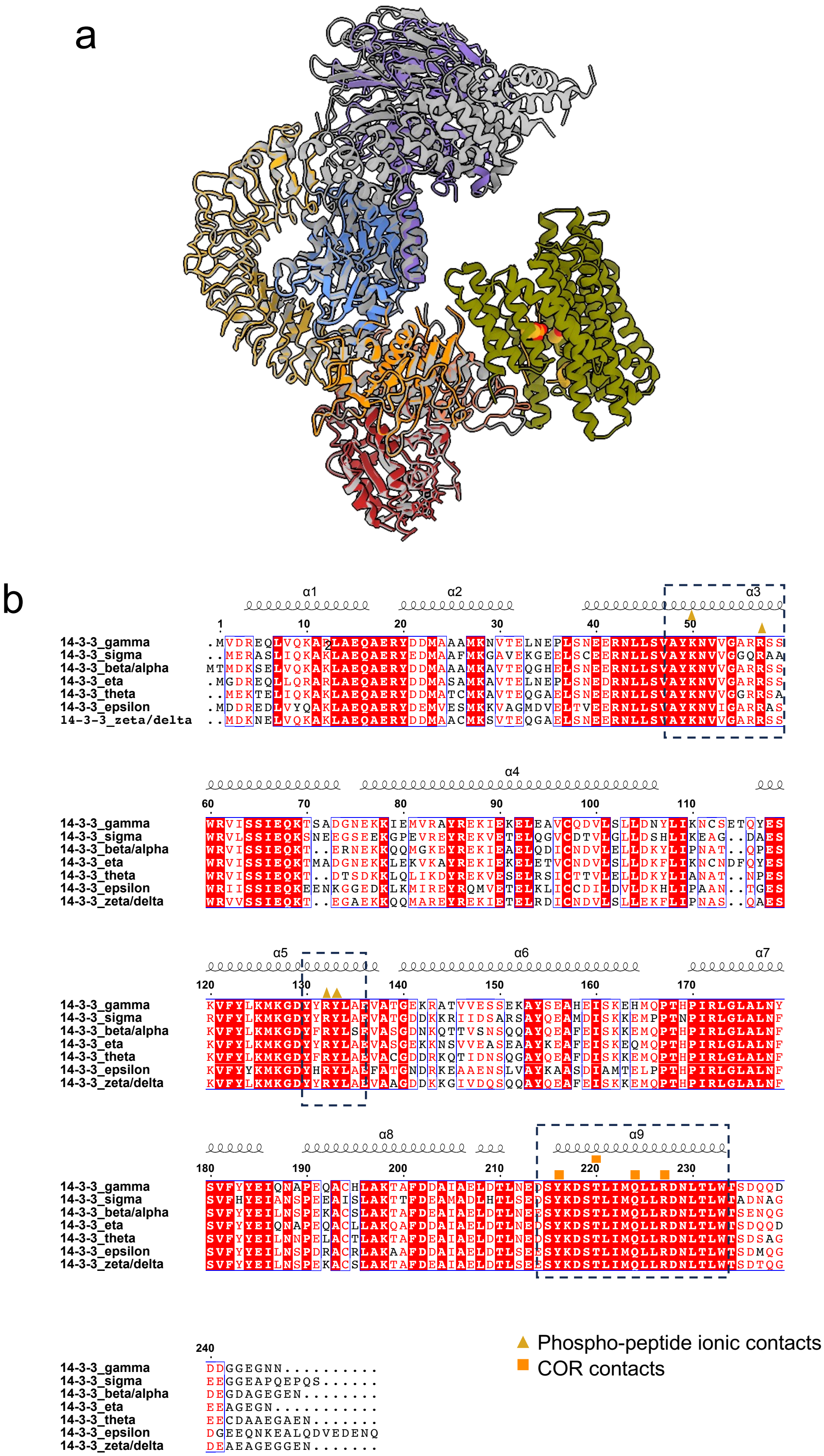
a
b
α1
α2
α3
α4
α5
α6
α7
α8
α9
14-3-3_gamma
14-3-3_sigma
14-3-3_beta/alpha
14-3-3_eta
14-3-3_theta
14-3-3_epsilon
14-3-3_zeta/delta
Phospho-peptide ionic contacts
COR contacts

Supplementary Fig. 7. Comparative structural and sequence analysis of LRRK2 interactions with 14-3-3. a. Superposition of the LRRK2:14-3-3_2 complex structure (colored as in Fig. 1) with the previously reported cryo-EM structure of the unliganded monomeric LRRK2 (PDB: 7LHW) shown in grey. **b.** Sequence alignment of human 14-3-3 isoforms. The secondary structure elements are indicated above the alignment. Identically conserved residues are shaded in red. Symbols above the alignment indicate 14-3-3 residues involved in the phospho-peptide ionic contacts (golden triangles) and COR contacts (orange squares). Dashed squares highlight these regions.

a

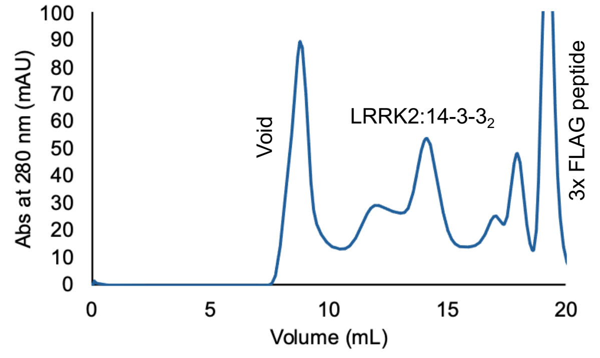

b

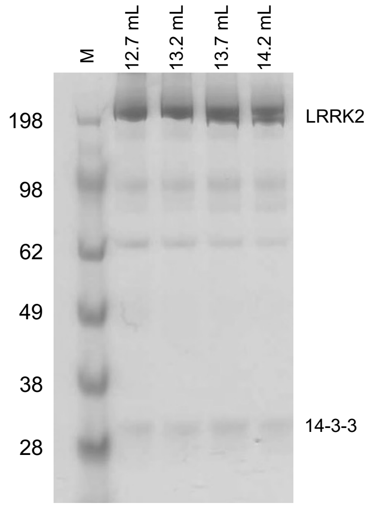

c

Accession/Gene	Protein	Coverage (%)	#PSMs	# Unique Peptides
Q5S007/LRRK2	Leucin-rich repeat serine/threonine-protein kinase 2	60%	3324	160
P61981/YWHAG	14-3-3 protein gamma	48%	203	17
P31946/YWHAB	14-3-3 protein beta/alpha	47%	163	16
Q04917/YWHAH	14-3-3 protein eta	41%	145	15
P63104/YWHAZ	14-3-3 protein zeta/delta	67%	144	17
P27348/YWHAQ	14-3-3 protein theta	31%	120	10
P62258/YWHAE	14-3-3 protein epsilon	50%	82	16

d

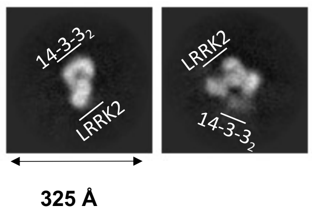

Supplementary Fig. 8. Isolation and characterization of human LRRK2/14-3-3 complex formed in a cellular context. a. Gel filtration chromatogram of the LRRK2/14-3-3 complex using a Superose 6 Increase 10/300 column. **b.** SDS-PAGE analysis confirms the co-elution and formation of the complex. **c.** Mass spectrometry validated the complex formation. Number of total (Peptide Spectrum Matches (PSMs) and unique peptides are reported. **d.** Representative 2D class averages from diagnostic cryo-EM of the LRRK2/14-3-3 complex. Refer to source data for complete membrane images and raw values.

a

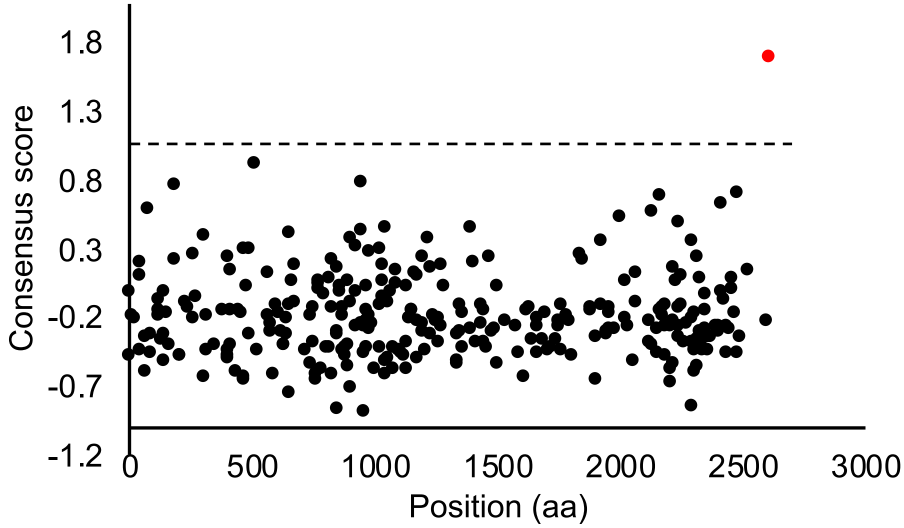

b

Confidence	Annotated Sequence	Modifications	# PSMs
High	[K].SAVEEGTASGSDGNFSEDVLSK.[F]	1xPhospho [S1/T]; 850	5
High	[R].YQMKSAVEEGTASGSDGNFSEDVLSK.[F]	1xPhospho [S3/T]; 860	13
High	[R].YQMKSAVEEGTASGSDGNFSEDVLSK.[F]	1xPhospho [S3/Y/T]; 860	13
High	[K].KKSNSISVGEFYR.[D]	1xPhospho [S2]; 910	12
High	[K].SNSISVGEFYR.[D]	1xPhospho [S2]; 910	56
High	[K].KSNSISVGEFYR.[D]	1xPhospho [S2]; 910	69
High	[R].HSNSLGPIFDHEDLLKR.[K]	1xPhospho [S2]; 935	64
High	[R].HSNSLGPIFDHEDLLK.[R]	1xPhospho [S2]; 935	109
High	[K].ILSSDDSLR.[S]	2xPhospho [S2; S3]; 955, 958	5
High	[K].ILSSDDSLR.[S]	1xPhospho [S2]; 955	41
High	[R].HSDSISSLASER.[E]	1xPhospho [S2]; 973	82
High	[R].HSDSISSLASEREYITSLDLSANELR.[D]	1xPhospho [S2]; 973	4
High	[R].HSDSISSLASEREYITSLDLSANELR.[D]	2xPhospho [S2; S3]; 973 975	2
High	[R].HSDSISSLASER.[E]	2xPhospho [S2; S4] 973, 976	16
High	[R].KILSSDDSLR.[S]	1xPhospho [S2]; 955	17
High	[K].FDEWTFIPDSSMDSVFAQSDDLDSEGSEGSFLVK.[K]	1xPhospho [S1]; 881	6
High	[K].FDEWTFIPDSSMDSVFAQSDDLDSEGSEGSFLVK.[K]	1xPhospho [S5]; 885	4
High	[R].CSPNLQR.[H]	1xPhospho [S2]; 926	30

Supplementary Fig. 9. Analysis of putative 14-3-3 binding sites in LRRK2. a. Prediction of 14-3-3 consensus binding sites in LRRK2, based on three different

classifiers (ANN, PSSM, and SVM) from the 14-3-3-Pred webserver[81]. Data points represent predicted binding sites in the LRRK2 sequence. The red point indicates the consensus score for a conventional binding motif (RSXSXP). No high-confidence binding motifs were identified in LRRK2 based on the consensus score. **b.** Identification of phospho-sites in purified LRRK2 protein in the LRRK2:14-3-3_2 complex by mass spectrometry. The number of peptides (Peptide Spectrum Matches (PSMs), and confidence in phosphorylation are reported. Refer to source data for raw values.

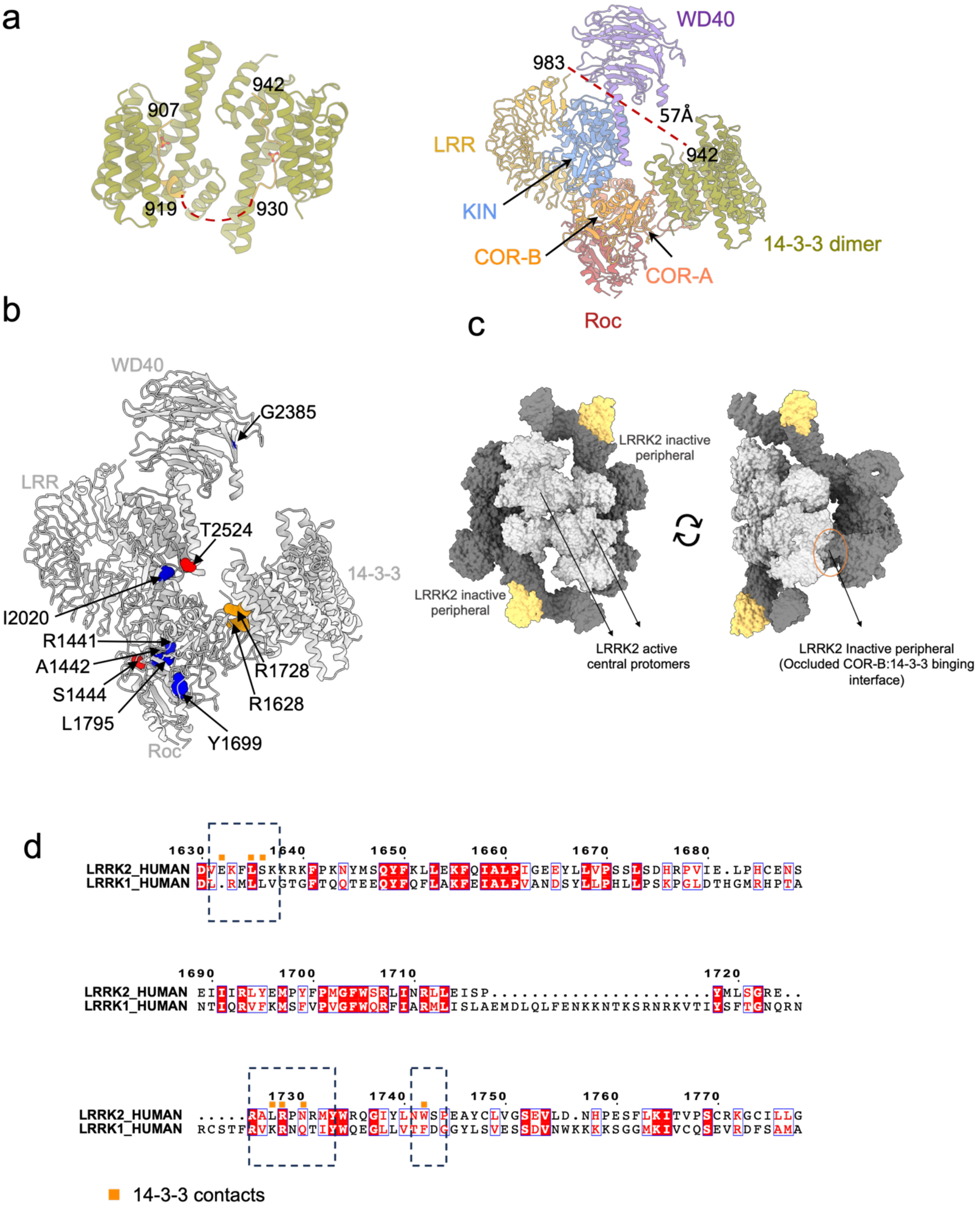

Supplementary Fig. 10. Additional structural analysis and hypothetical binding regions in the LRRK2:14-3-3$_2$ complex. a. Illustration of missing loop regions

connecting the 14-3-3-binding-phoshopeptides to the LRR domain and between these regions, depicted with dashed red lines. **b.** Mapping of additionally putative 14-3-3 phospho-binding sites previously reported[77] (shown in red). PD associated mutations located at (orange) and away from (blue) the 14-3-3 binding interface are also shown. **c.** Structural incompatibility of the LRRK2/14-3-3 interaction in the LRRK2 tetramer (PDB: 8FO9). The active central LRRK2 protomers are shown in light gray, the inactive peripheral LRRK2 protomers in dark gray, and Rab29 in yellow. **d.** Sequence alignment of the COR domain in human LRRK2 and LRRK1. Identically conserved residues are shaded in red. Symbols above the alignment indicate the 14-3-3 contacts (orange squares), with dashed squares highlighting these regions.

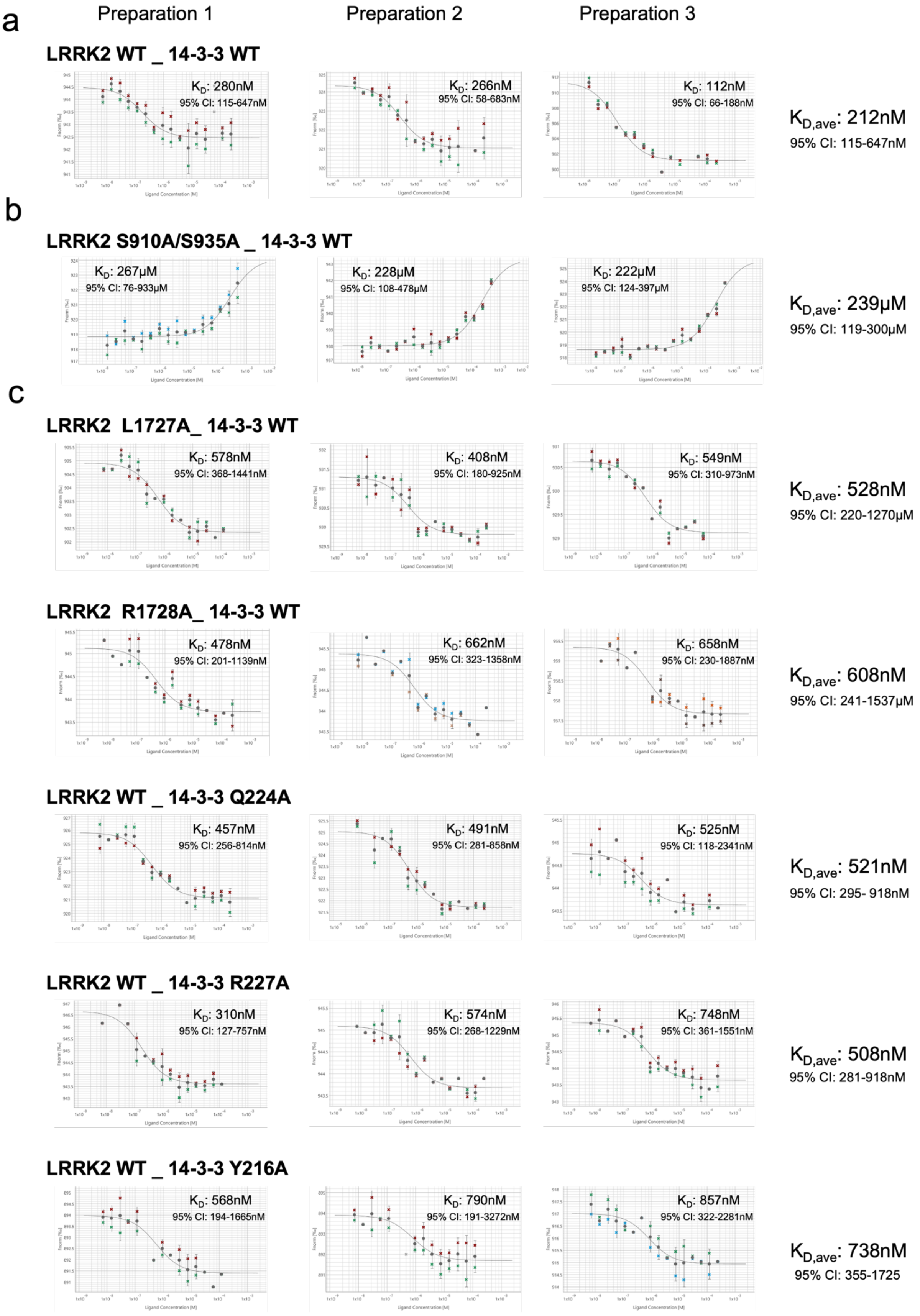

a
Preparation 1
Preparation 2
Preparation 3
LRRK2 WT _ 14-3-3 WT
KD: 280nM
95% CI: 115-647nM
KD: 266nM
95% CI: 58-683nM
KD: 112nM
95% CI: 66-188nM
KD,ave: 212nM
95% CI: 115-647nM
b
LRRK2 S910A/S935A _ 14-3-3 WT
KD: 267μM
95% CI: 76-933μM
KD: 228μM
95% CI: 108-478μM
KD: 222μM
95% CI: 124-397μM
KD,ave: 239μM
95% CI: 119-300μM
c
LRRK2 L1727A_ 14-3-3 WT
KD: 578nM
95% CI: 368-1441nM
KD: 408nM
95% CI: 180-925nM
KD: 549nM
95% CI: 310-973nM
KD,ave: 528nM
95% CI: 220-1270μM
LRRK2 R1728A_ 14-3-3 WT
KD: 478nM
95% CI: 201-1139nM
KD: 662nM
95% CI: 323-1358nM
KD: 658nM
95% CI: 230-1887nM
KD,ave: 608nM
95% CI: 241-1537μM
LRRK2 WT _ 14-3-3 Q224A
KD: 457nM
95% CI: 256-814nM
KD: 491nM
95% CI: 281-858nM
KD: 525nM
95% CI: 118-2341nM
KD,ave: 521nM
95% CI: 295- 918nM
LRRK2 WT _ 14-3-3 R227A
KD: 310nM
95% CI: 127-757nM
KD: 574nM
95% CI: 268-1229nM
KD: 748nM
95% CI: 361-1551nM
KD,ave: 508nM
95% CI: 281-918nM
LRRK2 WT _ 14-3-3 Y216A
KD: 568nM
95% CI: 194-1665nM
KD: 790nM
95% CI: 191-3272nM
KD: 857nM
95% CI: 322-2281nM
KD,ave: 738nM
95% CI: 355-1725
Ligand Concentration [M]
Fnorm [‰]

Supplementary Fig. 11. Binding affinity between LRRK2 and 14-3-3 determined by MST. MST was used to determine the binding affinities between LRRK2 and 14-3-3 using wild-type (a) or mutated proteins at residues located at the LRRK2:14-3-3_2 primary (b) or secondary (c) binding sites. Each binding curve was generated in duplicate for three independent preparations (n=3), the error bars represent mean ± SD. The 95% confidence intervals (CI) of the dissociation constants (K_D) are reported. Refer to Source data file for raw values.

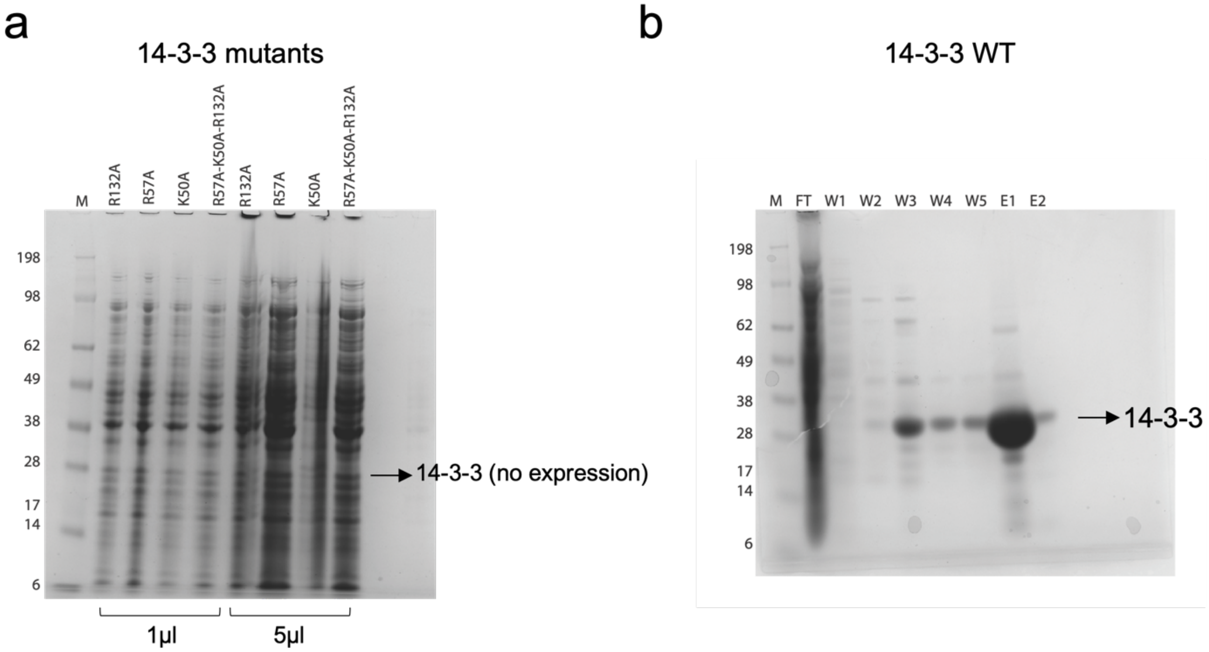

Supplementary Fig. 12. Expression analysis for 14-3-3 mutants. a. Representative small-scale expression trials of 14-3-3γ mutants harboring individual substitutions at residues R132, R57, and K50, as well as the triple mutant R57A/K50A/R132A, show poor or no detectable expression, suggesting that these mutations impact protein stability. **b.** A sample of purified WT 14-3-3γ is shown for reference to indicate the expected migration and purity. Note that the WT sample was obtained through a standard purification protocol and is not directly comparable to the mutant lysates in panel *a*. This figure is included to highlight the apparent loss of expression or solubility in the mutants, not for quantitative comparison. Refer to source data for complete membrane images.

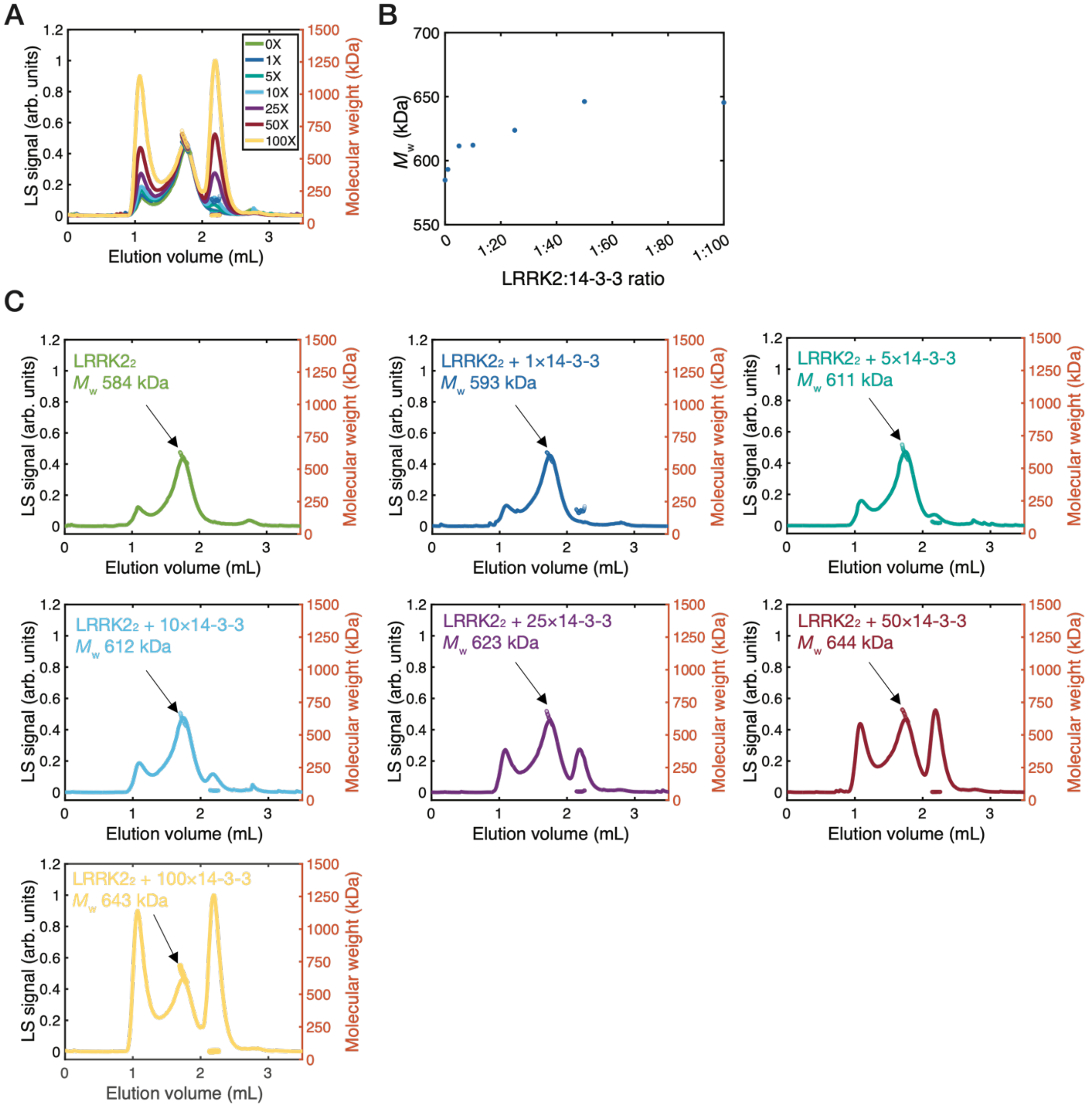

Supplementary Fig. 13. Interaction of LRRK2 dimer with 14-3-3. a. Overlay of the SEC-MALS chromatograms for the LRRK2 illustrating the effect of increasing concentrations of 14-3-3. **b.** Corresponding plot demonstrating a 14-3-3-concentration dependent increase in molecular weight, consistent with the binding of a single 14-3-3 dimer to a LRRK2 dimer which has a molecular weight of 616 KDa. **c.** Individual chromatograms corresponding to each 14-3-3 concentration, as shown in panel (a). Refer to source data for raw values.

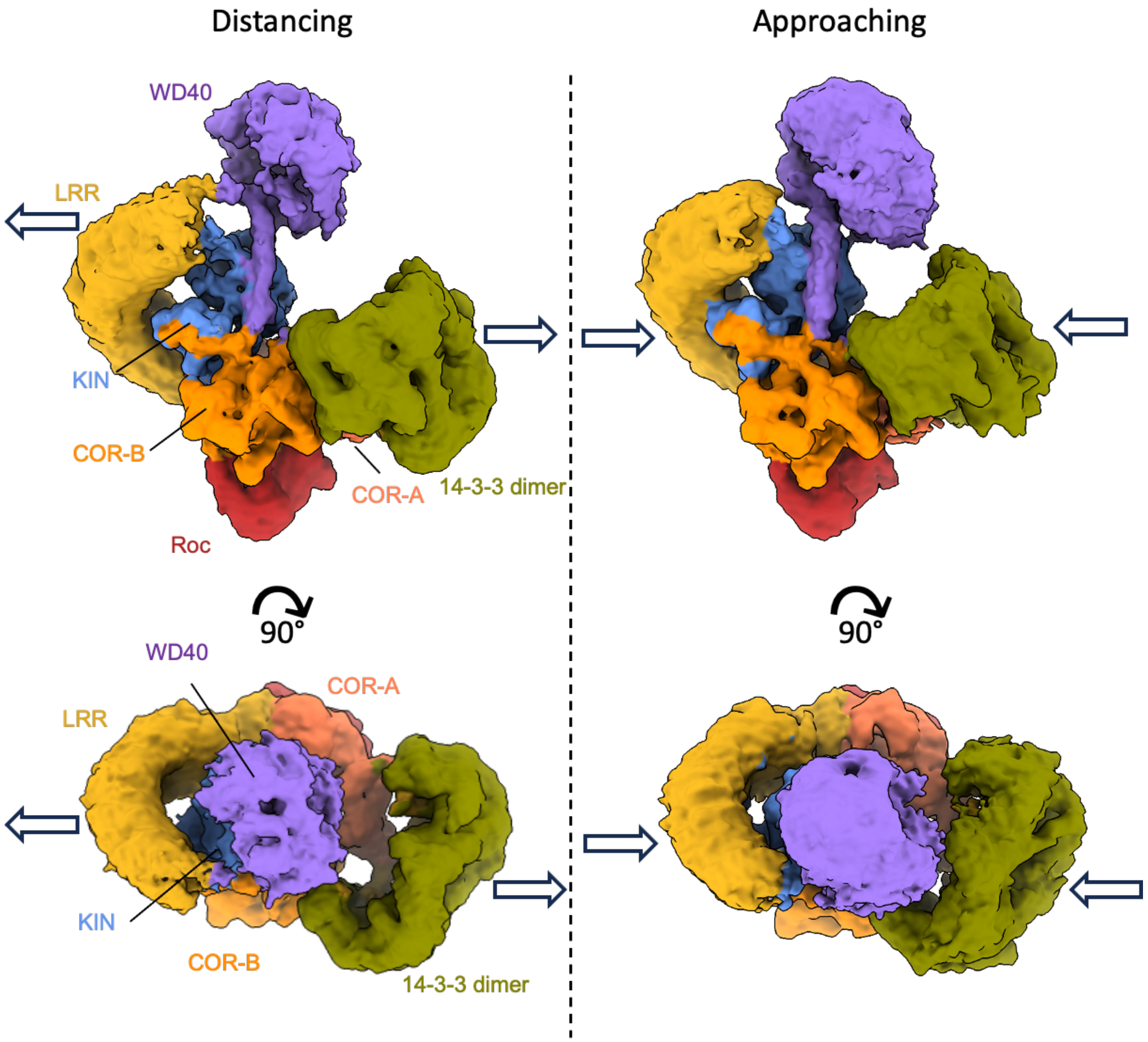

Supplementary Fig. 14. Dynamic Interactions in the LRRK2:14-3-3$_2$ complex revealed by 3D variability analysis (3DVA). 3DVA reveals synchronized movement of the LRR domain and 14-3-3 dimer relative to the Roc-COR-Kinase-WD40 portion of the protein. Arrows indicate the directions of motion captured by the 3D variability analysis. See also, Supplementary Movie 1. Figures and movie were generated using a filter resolution of 7 Å to enhance visualization of the dynamic modes.

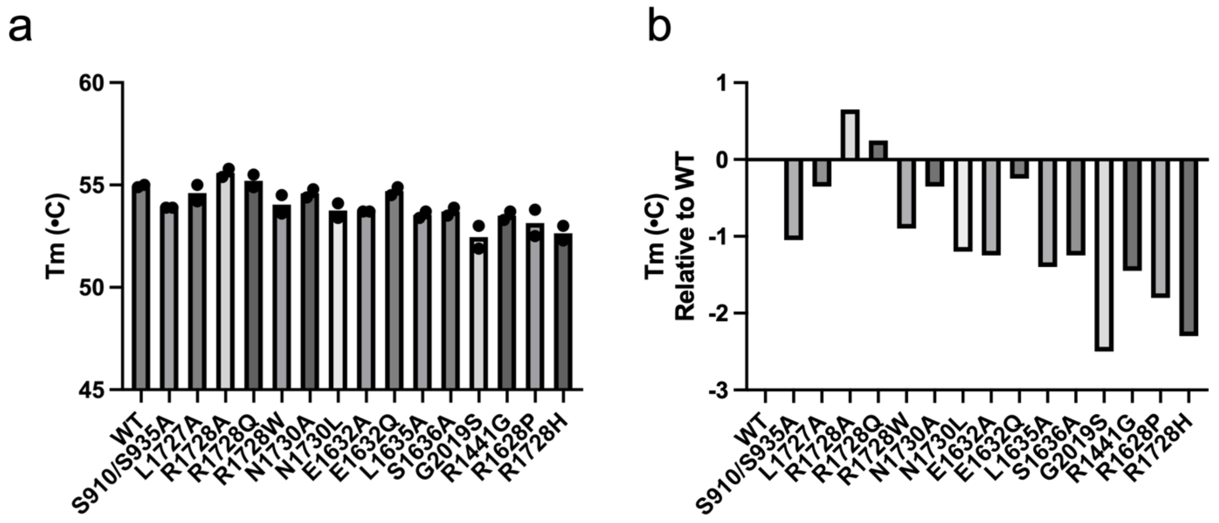

Supplementary Fig. 15. Thermal stability analysis of LRRK2 variants. a. Melting temperature (Tm) of LRRK2 variants including mutations at the LRRK2:14-3-3 interaction interface and other PD related mutations from two independent experiments. **b.** Comparison of melting temperatures across all tested LRRK2 variants, plotted relative to WT LRRK2. See Source data file for raw data.

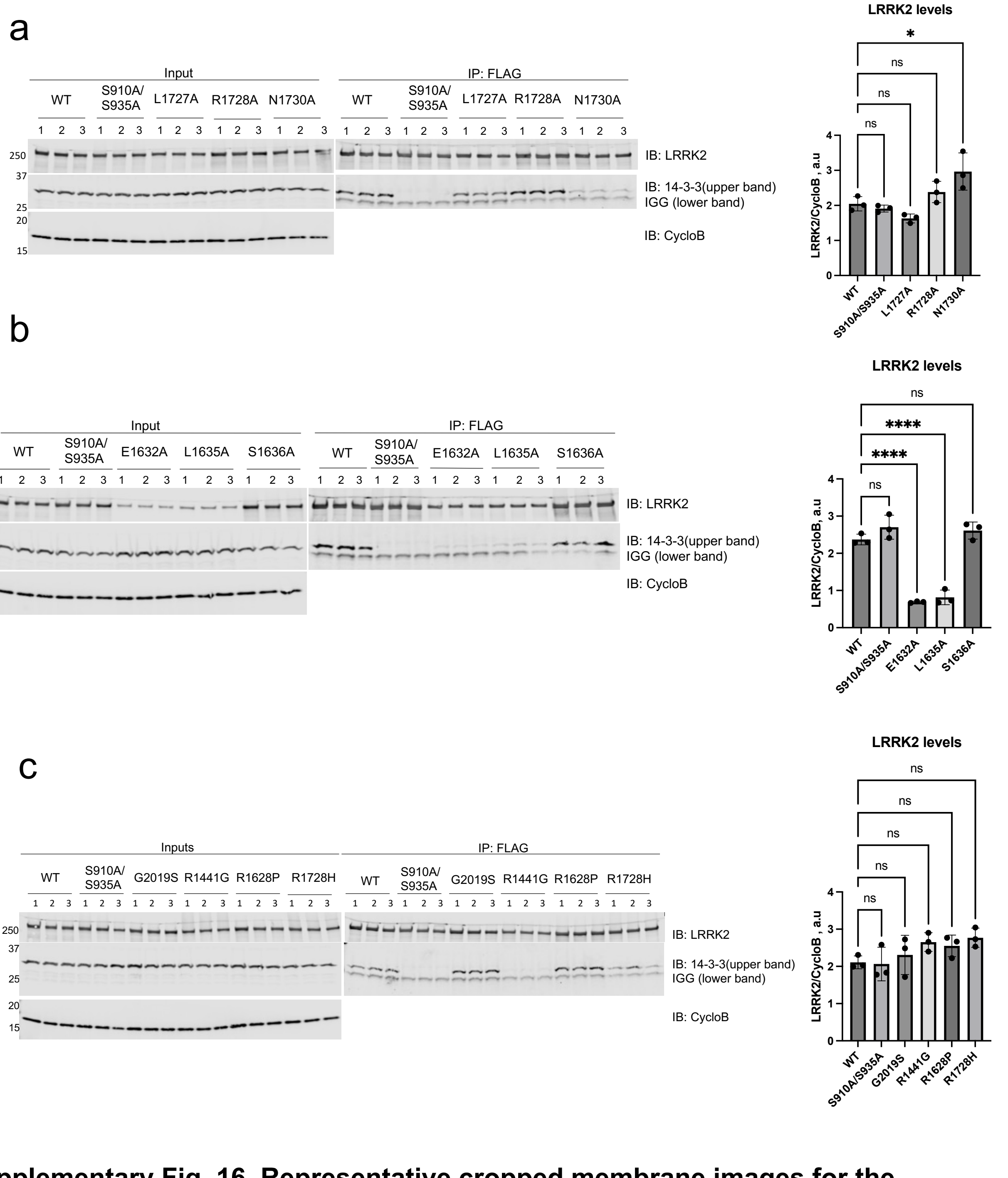

Supplementary Fig. 16. Representative cropped membrane images for the LRRK2/14-3-3 Co-IP experiments. a-b: Related to Fig. 2d, **c**: Related to Fig. 6a and 6c showing representative data for the results presented in these figure panels. LRRK2 levels were quantified from the Input samples, Data in a-c are mean ± SEM (n = 3 independent experiments). For full uncropped blot images, refer to the source data file.

See source data for significance of difference with the one-way Brown-Forsythe and Welch ANOVA test with the exact p values when applicable.

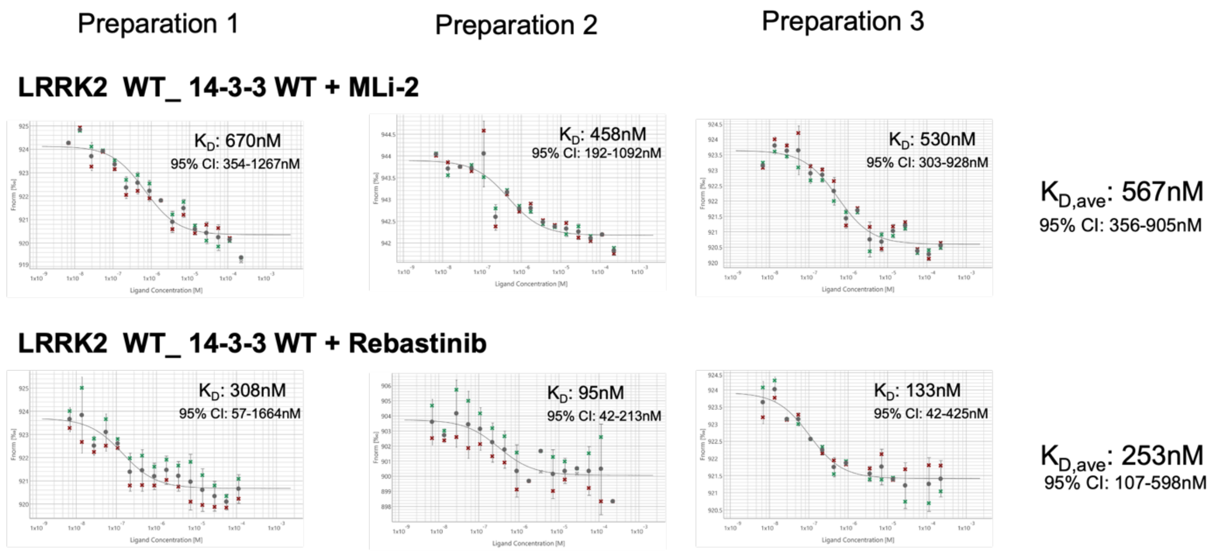

Supplementary Fig. 17. Binding affinity between LRRK2 and 14-3-3 determined by MST. MST was used to determine the binding affinities between LRRK2 and 14-3-3 using wild-type proteins in the presence of type I or type II inhibitors. Each binding curve was generated in duplicate for three independent preparations (n=3), the error bars represent mean ± SD. The 95% confidence intervals (CI) of the dissociation constants (K_D) are reported. Refer to Source data file for raw values.

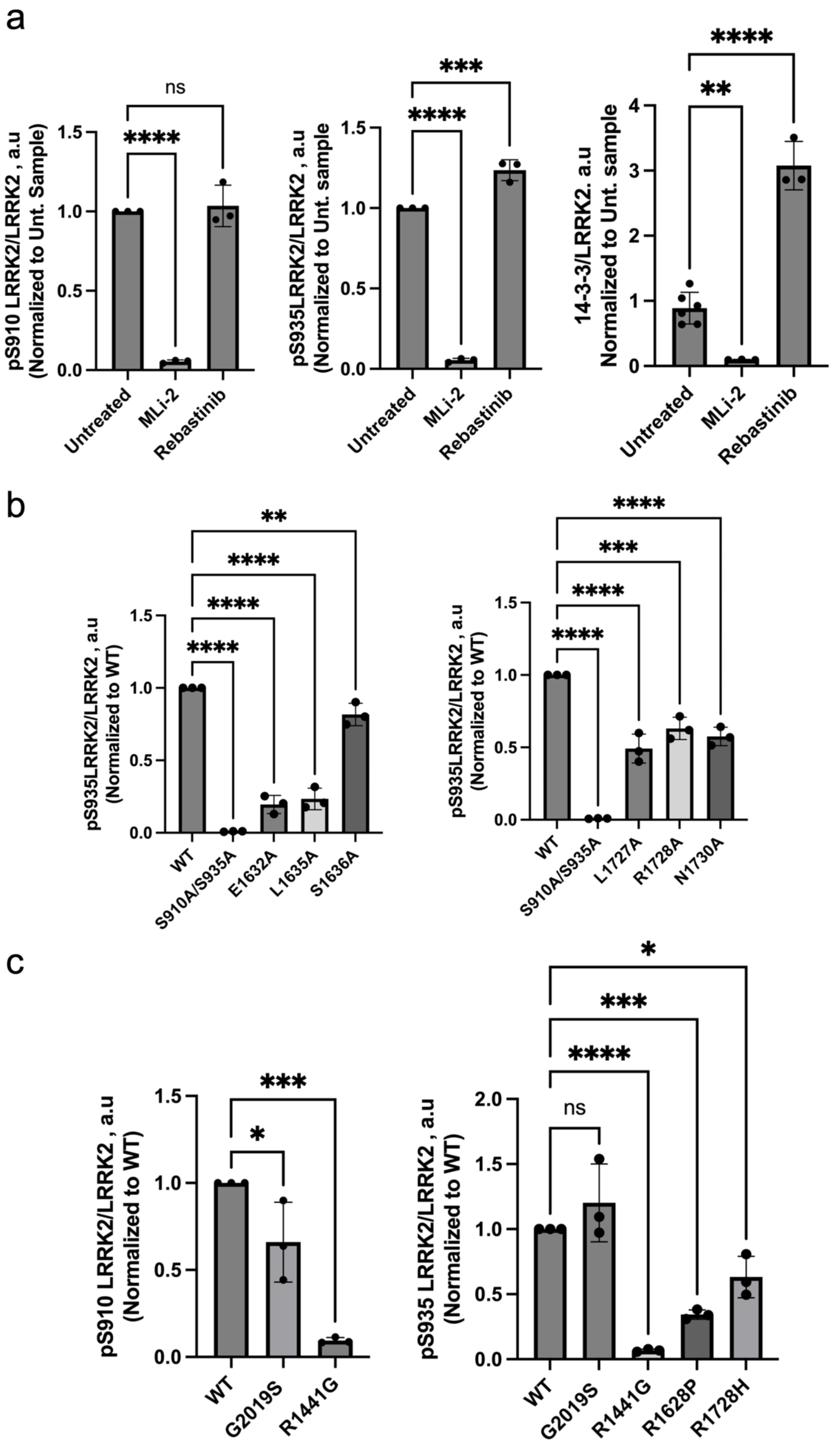
a
pS910 LRRK2/LRRK2 , a.u
(Normalized to Unt. Sample)
0.0
0.5
1.0
1.5
Untreated
MLi-2
Rebastinib
ns
****
pS935LRRK2/LRRK2 , a.u
(Normalized to Unt. sample
0.0
0.5
1.0
1.5
Untreated
MLi-2
Rebastinib
***
****
14-3-3/LRRK2. a.u
Normalized to Unt. sample
0
1
2
3
4
Untreated
MLi-2
Rebastinib
****
**
b
pS935LRRK2/LRRK2 , a.u
(Normalized to WT)
0.0
0.5
1.0
1.5
WT
S910A/S935A
E1632A
L1635A
S1636A
**
****
****
****
pS935LRRK2/LRRK2 , a.u
(Normalized to WT)
0.0
0.5
1.0
1.5
WT
S910A/S935A
L1727A
R1728A
N1730A
****
***
****
****
c
pS910 LRRK2/LRRK2 , a.u
(Normalized to WT)
0.0
0.5
1.0
1.5
WT
G2019S
R1441G
***
*
pS935 LRRK2/LRRK2 , a.u
(Normalized to WT)
0.0
0.5
1.0
1.5
2.0
WT
G2019S
R1441G
R1628P
R1728H
*
***
****
ns

Supplementary Fig. 18. Destabilization of the LRRK2/14-3-3 interaction leads to dephosphorylation of LRRK2 at the S910 and S935 sites in cells. a. Effects of LRRK2 kinase inhibitors on the phosphorylation levels at S910 (pS910) and S935 (pS935) (left) and on levels of co-immunoprecipitated 14-3-3 (right). **b**. Comparison of pS935 levels between WT LRRK2 and mutants in the COR-A (left) and COR-B (right) subdomains at the LRRK2:14-3-3_2 secondary interface. **c.** Comparison of pS910 (left) and pS935 (right) levels between WT LRRK2 and PD-associated mutations. Data in (a-c) are mean ± SEM (n = 3 independent experiments). See source data for membrane images and for significance of difference with the one-way Brown-Forsythe and Welch ANOVA test with the exact p values when applicable.

Supplementary Table 1. Reagent or Resource Table

REAGENT or RESOURCE	SOURCE	IDENTIFIER
**Antibodies**		
anti-LRRK2 primary antibody	Abcam	cat# ab133474
anti-Rab10 primary antibody	Abcam	cat# ab237703
anti-phospho Rab10 T73 primary antibody	Abcam	cat# ab241060
anti-14-3-3 gamma	Abcam	cat# 137048
goat anti-rabbit secondary antibody	LiCor	cat# 926-3221
anti-mouse secondary antibody IR-fluorescent	LiCor	cat# 926-68072
anti- pS910 LRRK2 antibody	Abcam	cat# ab133449
anti- pS935 LRRK2 antibody	Abcam	cat# ab133450
**Critical Commercial Assays and reagentes**		
Superose 6, 10/300 GL	Cytiva	cat# 29091596
Superdex 75 16/160 column	Cytiva	cat# 28-9893-33
Pierce Silver Stain Kit	Thermo Scientific	cat# 24612
Flag-M2 affinity resin	Sigma	cat# A2220
3×-Flag peptide	Genscript	cat# RP21087
Bac-to-Bac™ Baculovirus Expression System	Thermo Fisher	cat# 10359016
NEB Q5 Site-Directed Mutagenesis Kit	NEB	cat# E0554
0.22 µm Ultrafree-GV centrifugal filter	Sigma	cat# UFC30GVNB
**Deposited Data**		
LRRK2:14-3-3_2	This paper	Coordinates: PDB: 9CI3<br>Cryo-EM map: EMDB: EMD-45609
Coordinates of LRRK2 monomer	(Myasnikov, et al, 2021)	PDB: 7LHW
Coordinates of 14-3-3 gamma	(Papagrigoriou, et al., 2005)	PDB: 2B05
**Experimental Models: Cell lines and media**		
Expi 293F^{TM} cells (human)	Thermo Fisher Scientific	cat# A14527
Expi293^{TM} expression medium	Thermo Fisher Scientific	cat# A1435101

FreeStyle^{TM} 293 expression medium	Thermo Fisher Scientific	cat# 12338018
HEK293FT	ATCC	cat#PTA-5077, RRID:CVCL_6911
**Recombinant DNA**		
Flag-LRRK2-pFastBac1 for WT and mutants	This paper	N/A
14-3-3γ _pET21b for WT and mutants	This paper	N/A
rab10-pET21b	This paper	N/A
**Software and Algorithms**		
14-3-3-pred	Madeira, *et al*, 2015	https://www.compbio.dundee.ac.uk/1433pred/
ESPript 3.0	Robert, *et al*, 2014	https://espript.ibcp.fr/ESPript/cgi-bin/ESPript.cgi
ImageStudio Lite software, v.5.2.5	N/A	https://www.licor.com/bio/image-studio/
Prism, v.10.2.0	N/A	https://www.graphpad.com
Cryosparc	Punjani, *et al*, 2017	https://cryosparc.com
Topaz	Bepler *et al*, 2019,2020	https://github.com/tbepler/topaz
EPU	Thermo Fisher	https://www.thermofisher.com/us/en/home/electron-microscopy/products/software-em-3d-vis/epu-software.html
deepEMhancer v0.13	Sanchez-Garcia, *et al*, 2021	https://github.com/rsanchezgarc/deepEMhancer
USF chimera	Pettersen, *et al*, 2004	https://www.cgl.ucsf.edu/chimera/
Coot	Emsley and Cowtan, 2004	https://www2.mrc-%20lmb.cam.ac.uk/personal/pemsley/coot
Phenix	Adams, *et al*., 2010	https://phenix-online.org
**Other**		
R1.2/1.3 300 mesh Au holey carbon grids	Electron Microscopy Sciences	Cat# Q350AR1.3

Supplementary Table 2. Cryo-EM data collection, Refinement and Validation Statistics

	**LRRK2:14-3-3$_2$**
**Data collection**	
Microscope	Talos Arctica
Camera	K3
Magnification	100k, EFTEM mode
Voltage (kV)	200
Electron exposure (e^-/Å^2)	50
Number of frames collected per micrograph	50
Energy filter slit width	20eV
Defocus range (μm)	-0.8 to -2.5
Pixel size (Å)	0.81
Movies (no.)	152,529
Initial particle images (no.)	6,317,931
Final particle images (no.)	432,285
Map resolution (Å)	3.96
FSC threshold	0.132
Map sharpening B factor (Å^2)	171.6
EMDB code	
**Model building and refinement**	
Initial model used (PDB)	7LHW, 2B05
Model composition	
Non-hydrogen atoms	15949
Protein residues	2002
Protein molecules	3
Real-space correlation	
CCvolume	0.58
CCmask	0.58
Mean B factor (Å^2)	55.65
RMS deviations	
Bond lengths (Å) (outliers >4σ)	0.003 (0)
Bond angles (°) (outliers >4σ)	0.777 (16)
Validation	
MOLProbity score	2.34
Clashscore	19.16
Rotamer outliers (%)	1.30
CaBLAM outliers (%)	0.20
Cβ outliers (%)	0.00
Ramachandran plot	
Favored (%)	92.32
Allowed (%)	7.63
Outliers (%)	0.05
PDB code	9CI3
