# Supplementary material for: 14-3-3 binding maintains the Parkinson’s associated kinase LRRK2 in an inactive state
Source: Nat Commun. 2025 Aug 5;16:7226. doi: 10.1038/s41467-025-62337-1 (PMC12325948; doi:10.1038/s41467-025-62337-1)
Supplement: Supplementary file 2 — Description of Additional Supplementary Files [file 41467_2025_62337_MOESM2_ESM.pdf]

Description of Additional Supplementary Files

File name: Supplementary Movie 1

Description: 3D Variability Analysis (3DVA) of the LRRK2:14-3-3_2 complex reveals synchronized motion of the LRR domain and the 14-3-3 dimer relative to the Roc-COR-KinaseWD40 core. The movie was generated using a filtered cryo-EM map at 7 Å resolution to enhance visualization of dynamic modes, consistent with the structural interpretation shown in Supplementary Fig. 14.
