# Supplementary material for: 14-3-3 binding maintains the Parkinson’s associated kinase LRRK2 in an inactive state
Source: Nat Commun. 2025 Aug 5;16:7226. doi: 10.1038/s41467-025-62337-1 (PMC12325948; doi:10.1038/s41467-025-62337-1)
Supplement: Supplementary file 4 — Reporting Summary [file 41467_2025_62337_MOESM4_ESM.pdf]

nature portfolio

Corresponding author(s): Ping Zhang

Last updated by author(s): May 30, 2025

Reporting Summary

Nature Portfolio wishes to improve the reproducibility of the work that we publish. This form provides structure for consistency and transparency in reporting. For further information on Nature Portfolio policies, see our Editorial Policies and the Editorial Policy Checklist.

Statistics

For all statistical analyses, confirm that the following items are present in the figure legend, table legend, main text, or Methods section.

n/a	Confirmed	
☐	☒	The exact sample size (*n*) for each experimental group/condition, given as a discrete number and unit of measurement
☐	☒	A statement on whether measurements were taken from distinct samples or whether the same sample was measured repeatedly
☐	☒	The statistical test(s) used AND whether they are one- or two-sided<br>*Only common tests should be described solely by name; describe more complex techniques in the Methods section.*
☒	☐	A description of all covariates tested
☐	☒	A description of any assumptions or corrections, such as tests of normality and adjustment for multiple comparisons
☐	☒	A full description of the statistical parameters including central tendency (e.g. means) or other basic estimates (e.g. regression coefficient) AND variation (e.g. standard deviation) or associated estimates of uncertainty (e.g. confidence intervals)
☐	☒	For null hypothesis testing, the test statistic (e.g. *F*, *t*, *r*) with confidence intervals, effect sizes, degrees of freedom and *P* value noted<br>*Give P values as exact values whenever suitable.*
☒	☐	For Bayesian analysis, information on the choice of priors and Markov chain Monte Carlo settings
☒	☐	For hierarchical and complex designs, identification of the appropriate level for tests and full reporting of outcomes
☒	☐	Estimates of effect sizes (e.g. Cohen's *d*, Pearson's *r*), indicating how they were calculated

Our web collection on statistics for biologists contains articles on many of the points above.

Software and code

Policy information about availability of computer code

Data collection	EPU was used for cryo-EM data collection
Data analysis	Cryosparc 4.5, COOT 0.9.6, PHENIX 1.17.1, Chimera 1.13.1, were used for the analysis of cryo-EM data and generation of structural figures. Prism 10 was used for the analysis of co-IP, kinase and inhibition assays

For manuscripts utilizing custom algorithms or software that are central to the research but not yet described in published literature, software must be made available to editors and reviewers. We strongly encourage code deposition in a community repository (e.g. GitHub). See the Nature Portfolio guidelines for submitting code & software for further information.

Data

Policy information about availability of data

All manuscripts must include a data availability statement. This statement should provide the following information, where applicable:

- Accession codes, unique identifiers, or web links for publicly available datasets
- A description of any restrictions on data availability
- For clinical datasets or third party data, please ensure that the statement adheres to our policy

Three-dimensional cryo-EM density map has been deposited in the Electron Microscopy data Bank under accession number EMD-45609 . The coordinates of atomic models have been deposited in the Protein Data Bank under accession numbers PDB ID: 9CI3

Field-specific reporting

Please select the one below that is the best fit for your research. If you are not sure, read the appropriate sections before making your selection.

[x] Life sciences   [ ] Behavioural & social sciences   [ ] Ecological, evolutionary & environmental sciences

For a reference copy of the document with all sections, see nature.com/documents/nr-reporting-summary-flat.pdf

Life sciences study design

All studies must disclose on these points even when the disclosure is negative.

Sample size	Biochemical and cellular data were from at least duplicate independent experiments. The sample sizes used were sufficient to represent the reproducibility of the assays.
Data exclusions	There were no data exclusions.
Replication	The biochemical and cellular experiments were reproducible. The number of replications can be found in the figure legends and Data source file.
Randomization	It was unnecessary to perform randomized experiments in the current study.
Blinding	It was unnecessary to perform blinded experiments in the current study.

Reporting for specific materials, systems and methods

We require information from authors about some types of materials, experimental systems and methods used in many studies. Here, indicate whether each material, system or method listed is relevant to your study. If you are not sure if a list item applies to your research, read the appropriate section before selecting a response.

Materials & experimental systems

n/a	Involved in the study	
[ ]	[x]	Antibodies
[ ]	[x]	Eukaryotic cell lines
[x]	[ ]	Palaeontology and archaeology
[x]	[ ]	Animals and other organisms
[x]	[ ]	Human research participants
[x]	[ ]	Clinical data
[x]	[ ]	Dual use research of concern

Methods

n/a	Involved in the study	
[x]	[ ]	ChIP-seq
[x]	[ ]	Flow cytometry
[x]	[ ]	MRI-based neuroimaging

Antibodies

Antibodies used	anti-LRRK2 primary antibody Abcam cat# ab133474, anti-Rab10 primary antibody Abcam cat# ab237703, anti-phospho Rab10 T73 primary antibody Abcam cat# ab241060, anti-14-3-3 gamma Abcam cat# 137048, goat anti-rabbit secondary antibody LiCor cat# 926-3221, and anti-mouse secondary antibody IR-fluorescent LiCor cat# 926-68072, anti-LpS910-LRRK2 primary antibody Abcam cat# ab133449, anti-LpS935-LRRK2 primary antibody Abcam cat# ab133450
Validation	Validation of the antibodies in terms of species recognition and application was obtained from the supplier's data sheets. The supplier and RRID numbers for the antibodies are listed in Supplementary Table 1.

Eukaryotic cell lines

Policy information about cell lines

Cell line source(s)	HEK-293FT, Expi-293F, cells were obtained from Thermofisher.
Authentication	Cell lines were obtained from Thermofisher and not independently authenticated.
Mycoplasma contamination	All cell lines tested negative for mycoplasma.
Commonly misidentified lines (See ICLAC register)	No misidentified lines were used.
